# Beyond Genetics: Exploring Lifestyle, Microbiome, and Social Determinants in Oral Cancer Development

**DOI:** 10.3390/cancers17071094

**Published:** 2025-03-25

**Authors:** Anil Menon, Vimi S. Mutalik, Yongqiang Chen, SPD. Ponamgi, Sujatha Peela, Robert J. Schroth, Saeid Ghavami, Prashen Chelikani

**Affiliations:** 1 Department of Preventive Dental Sciences, Dr. Gerald Niznick College of Dentistry, University of Manitoba, Winnipeg, MB R3E 0W2, Canada; anil.menon@umanitoba.ca (A.M.); robert.schroth@umanitoba.ca (R.J.S.); 2 Department of Dental Diagnostics and Surgical Sciences, Dr. Gerald Niznick College of Dentistry, University of Manitoba, Winnipeg, MB R3E 0W2, Canada; vimi.mutalik@umanitoba.ca; 3 Manitoba Chemosensory Biology Research Group, Department of Oral Biology, Dr. Gerald Niznick College of Dentistry, University of Manitoba, Winnipeg, MB R3E 0W2, Canada; yongqiang.chen@umanitoba.ca; 4 Department of Biotechnology, Andhra University College of Science and Technology, Andhra University, Visakhapatnam 530003, Andhra Pradesh, India; prof.dhanunjay@andhrauniversity.edu.in; 5 Department of Biotechnology, Dr. B. R Ambedkar University, Srikakulam 532410, Andhra Pradesh, India; drpsujatha@brau.edu.in; 6 Department of Human Anatomy and Cell Sciences, Max Rady College of Medicine, University of Manitoba, Winnipeg, MB R3E 0J9, Canada; saeid.ghavami@umanitoba.ca

**Keywords:** oral cancer, socioeconomic status, diet, microbiome, lifestyle, oral squamous cell carcinoma, mouth, tea, coffee, fish

## Abstract

Oral cancer refers to cancers originating in the oral cavity and oropharyngeal regions. It is the 16th most prevalent cancer and the sixth leading cause of cancer-related deaths. However, the mechanisms of its progression are still being understood, and interventions to provide early diagnosis need to be improved. More studies have recently been conducted on oral cancer, and many reviews have summarized the findings in this field, focusing on individual factors. However, few review articles have evaluated the combinational impacts of different factors on oral cancer. This review aimed to provide an overview of the combinational effects of three extracellular factors, including lifestyle habits, oral microbiome, and socioeconomic factors, on oral cancer progression. Oral cancer is differentially affected by lifestyle habits; high-sugar diets, processed foods, alcohol, smoking, and possibly sleep disorders benefit its progression, whereas eating natural diets, such as fruits, vegetables, fish, and garlic, drinking tea or coffee, and physical exercise can combat it. The oral microbiome could suppress or promote oral cancer progression. Low socioeconomic status can impact oral cancer development. Furthermore, crosstalk among these three factors affects oral cancer progression. This review has limitations in not including all oral cancer-affecting factors and all important publications. More focus should be placed on the combinational effects of multiple factors on oral cancer progression and treatment. The findings in this study could update researchers on the landscape of oral cancer progression and help formulate approaches to promote oral cancer prevention and treatment.

## Simple Summary

The global health challenge of oral cancer extends beyond genetic factors to include various other influences. This review studies the effects of interactions between lifestyle behaviors, such as diet choices and substance use, along with oral microbiome composition and socioeconomic influences, on oral cancer progression. Dietary patterns high in sugar and processed foods may raise the risk of oral cancer, whereas diets with fruits, vegetables, fish, green tea, and coffee show potential protective effects. The oral microbiome serves a dual purpose as it can both advance and defend against cancer development. Poor socioeconomic status leads to restricted healthcare access, which results in delayed cancer detection and more severe health outcomes. This review shows how multiple factors interact to suggest effective prevention approaches and targeted therapies, which could lead to better worldwide oral cancer treatment results and patient survival rates.

## 1. Introduction

Cancer poses a significant challenge as a societal, public health, and economic issue in the 21st century, accounting for nearly one in six deaths (16.8%) in general and approximately one in four deaths (22.8%) among noncommunicable diseases [1]. Oral cancer refers to malignancies originating in the oral cavity and oropharyngeal regions, predominantly comprising oral squamous cell carcinoma (OSCC), which accounts for over 90% of cases [2]. It commonly affects the tongue, lips, buccal mucosa, gingiva, and floor of the mouth [3]. Oral cancer is also classified as the primary subtype of head and neck squamous cell carcinomas (HNSCCs) [2,4]. Based on the Global Cancer Observatory, HNSCCs are the seventh most common type of cancer, accounting for approximately 890,000 new cases, of which 380,000 are from the lip and oral cavity [4]. Globally, oral cancer ranks as the 16th most prevalent cancer and remains the sixth leading cause of cancer-related deaths [5,6]. In 2022, there were 389,485 new cases of oral cancer worldwide [7], with India bearing the highest oral cancer burden [8]. However, the mechanisms underlying the progression of oral cancer are still being understood, and interventions for an early diagnosis and its treatment need to be improved.

In recent years, many studies have investigated pathways to the development of oral cancer and its treatment. Many review articles have summarized the research in these aspects, such as epidemiology, diagnostics, and therapy of oral cancer [7], roles of oral cancer stem cells in treatment resistance and tumor recurrence [9], the chemoprevention of natural products against oral cancer [10], the benefits of oral cancer screening [11], the roles of non-coding ribonucleic acids (RNAs) in oral cancer [12], the roles of autophagy in oral cancer [13], the roles of cell death pathways in oral cancer [14], the impact of oral microbiome dysbiosis on oral cancer [15], the roles of plant-based diets in oral cancer prevention and therapy [16], the impacts of oxidative stress in oral cancer pathogenesis [17], risk prediction models of oral cancer [18], the effects of tea and coffee on oral cancer risk [19], the impact of genetics and epigenetics on the initiation, progression, and metastasis of oral cancer [20], and the link between oral cancer and diabetes [21], microRNAs [22], and exosomes [23]. However, the combinational impacts of different factors on oral cancer have rarely been reviewed in the literature. This review aims to provide an overview of the roles of three extracellular factors, including lifestyles, oral microbiome, and socioeconomic factors, in oral cancer progression, which reflect at least most of the extracellular factors studied in the literature. It could update the knowledge in these fields and provide insight into developing strategies to enhance the early diagnosis, management, and treatment of oral cancer.

## 2. Oral Cancer and the Underlying Cellular Mechanisms

### 2.1. Oral Cancer

**Figure 1:**
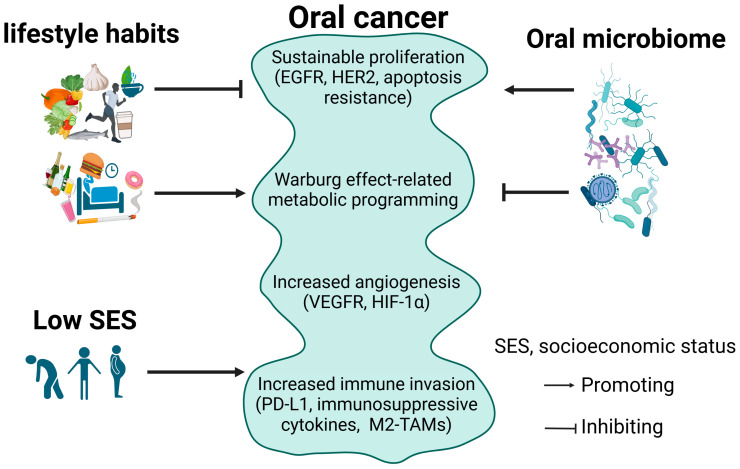
Oral cancer progression is affected by different factors. EGFR, epidermal growth factor receptor; HER2, human epidermal growth factor receptor 2; VEGFR, vascular endothelial growth factor receptor; HIF-1α, hypoxia-inducible factor-1α; PD-L1, programmed cell death ligand 1; M2-TAMs, M2-like tumor-associated macrophages.

Oral cancer comprises histologically and molecularly diverse malignancies, including OSCC, salivary gland carcinoma, odontogenic carcinoma, and oral mucosal melanoma [24,25]. The most common oral cancer is OSCC, which involves genetic mutations in genes like tumor protein p53 (*TP53*) and cyclin-dependent kinase inhibitor 1A (*CDKN2A*) that cause abnormal cell-cycle regulation and apoptosis resistance [26]. Salivary gland carcinomas have very different molecular signatures, including fusion genes such as myeloblastosis viral oncogene homolog–nuclear factor I/B (*MYB-NFIB*), which may promote aggressive tumor growth [27]. Odontogenic carcinoma and mucosal melanomas are uncommon. They carry mutations in genes like catenin beta 1 (*CTNNB1*), adenomatous polyposis coli (*APC*), V-Raf murine sarcoma viral oncogene homolog B (*BRAF*), and neuroblastoma RAS viral oncogene homolog (*NRAS*), fueling oncogenic cascades [28]. The following hallmark features and gene signatures can help identify oral cancer. Oral cancer is characterized by the following features (Figure 1).

### 2.2. Sustained Proliferation in Oral Cancer

Intense proliferative processes involve the overexpression of growth factor receptors, such as epidermal growth factor receptor (EGFR) and human epidermal growth factor receptor 2 (HER2), and the proliferation marker Ki-67, which are hallmarks of oral cancer development [29]. Apoptosis resistance often results from mutations in tumor suppressors, such as TP53, and the overexpression of anti-apoptotic proteins, such as B-cell lymphoma 2 (BCL-2) [30,31].

### 2.3. Metabolic Reprogramming in Oral Cancer

Metabolic reprogramming is a hallmark of cancer, allowing tumor cells to keep proliferation high by fulfilling their large energy and biosynthetic needs [32,33]. It allows cancer cells to maintain a glycolytic energy supply even in oxygen-deprived conditions (the Warburg effect) [34]. In OSCC, the Warburg effect dominates, with cancer cells relying more on glycolysis than oxidative phosphorylation, even in the presence of oxygen; the metabolic switch permits the synthesis of rapidly occurring adenosine triphosphate (ATP) and intermediates required for nucleotide, lipid, and protein synthesis [35,36]. The upregulation of important glycolytic enzymes, including hexokinase 2 (HK2) and pyruvate kinase M2 (PKM2), contributes to the aggressive feature of OSCC [37]. Glycolysis-induced lipid deposition leads to a highly acidic tumor microenvironment (TME), fostering invasion and immune escape [33,38]. The metabolic reprogramming of the glands in saliva is observed in salivary gland carcinomas such as mucoepidermoid and adenoid cystic carcinomas [39]. These tumors promote glucose absorption through glucose transporter type 1 overexpression, increasing glycolysis flow. Lactate dehydrogenase-A (LDH-A) converts pyruvate to lactate and is often upregulated in tumor cells, allowing for long-term energy generation under hypoxia [40]. This metabolic adaptation facilitates tumor cell proliferation and spread.

Mucosal melanomas are highly metabolically plastic and thrive in nutrient-poor and oxygen-poor conditions. They can switch from glycolysis to oxidative phosphorylation in microenvironmental conditions. Hypoxia-inducible factor-1α (HIF-1α) and hypoxia-inducible factor-2α (HIF-2α) drive glycolytic gene expression under reduced oxygen conditions to maintain tumor cell survival in hostile environments [41,42]. These metabolic adaptations represent a promising therapeutic option for many types of oral cancers by targeting metabolic enzymes.

### 2.4. Angiogenesis in Oral Cancer

Angiogenesis refers to the growth of new blood vessels; it keeps tumors growing, delivers nutrients, and allows them to spread [43]. In OSCC, neovascularization is stimulated by the release of vascular endothelial growth factor (VEGF) and other pro-angiogenic molecules. Increased VEGF expression is linked to increased microvascular density, tumor growth, and adverse outcomes [44]. OSCC cells also show increased expression of angiopoietins and matrix metalloproteinases (MMPs), which break down the extracellular matrix to enable endothelial cells to move around and form vessels [45]. Salivary gland carcinomas, such as adenoid cystic carcinoma, have distinctive angiogenic profiles attributed to their invasive nature. These tumors often undergo perineural invasion aided by local angiogenesis [46]. Vascular growth has been linked to increased basic fibroblast growth factor (bFGF) and platelet-derived growth factor (PDGF) in these cancers. Moreover, HIF-1α activation during oxygen deprivation also drives angiogenic signaling [47]. Mucosal melanomas are extensively vascularized due to angiogenic factors like VEGF, bFGF, and interleukin-8 (IL-8). These molecules promote endothelial cell proliferation and vascular opening, permitting metastatic dissemination. EGFR or HER2 might not affect oral cancer angiogenesis because their common inhibitor, lapatinib, does not inhibit angiogenesis in HNSCC cells [48]. The interaction between angiogenesis and immune suppression also permits immune evasion, forming the aggressive clinical profile of mucosal melanomas. Targeting angiogenic signals can be used as a therapeutic strategy in these multiple oral cancer subtypes [47].

### 2.5. Immune Evasion in Oral Cancer

Immune evasion is a hallmark of oral cancer, in which tumor cells bypass immune surveillance to form long-term tumors [49]. Cancer cell immune evasion is accomplished by activating immune checkpoint proteins, such as programmed cell death ligand 1 (PD-L1), which deactivates T-cells [50]. The immune checkpoint proteins, including PD-L1, are commonly overexpressed in OSCC [50]. PD-L1 binds to programmed cell death protein 1 (PD-1) on T cells, inhibiting their cytotoxic effects and facilitating an immunosuppressive TME [51,52]. A high level of PD-L1 expression is associated with more advanced tumor status and poor clinical performance in patients with OSCC [53]. OSCC cells can produce immunosuppressive cytokines such as transforming growth factor-beta (TGF-β) and interleukin-10 (IL-10), which can, in turn, reduce anti-tumor immunity [53]. Salivary gland carcinomas, such as adenoid cystic carcinoma, establish an immunosuppressive environment by attracting regulatory T cells and myeloid-derived suppressor cells (MDSCs) [54]. These immune cells suppress effector T cell responses and secrete immunosuppressants that protect cancer cells from immune damage. The tumor-associated macrophages (TAMs) within these tumors adopt an anti-tumorigenic M2 morphology, promoting immune escape and tumor survival [55]. Mucosal melanomas often exhibit low levels of tumor-infiltrating lymphocytes (TILs), indicating an immune “cold” microenvironment [56]. Low immune infiltration correlates with failure to respond to immunotherapies [56]. Tumor cells could suppress major histocompatibility complex (MHC) molecules, diminishing antigen delivery and thereby preventing immune activation [57]. The insight into these immunosuppressive processes offers a basis for creating new immunotherapies for the oral cancer subtypes.

Knowledge of these hallmark features opens the door for targeted therapies, such as EGFR inhibitors, immune checkpoint blockers, and anti-angiogenic agents, to guide advances in oral cancer management [58].

## 3. Lifestyle Habits and Oral Cancer

**Figure 2:**
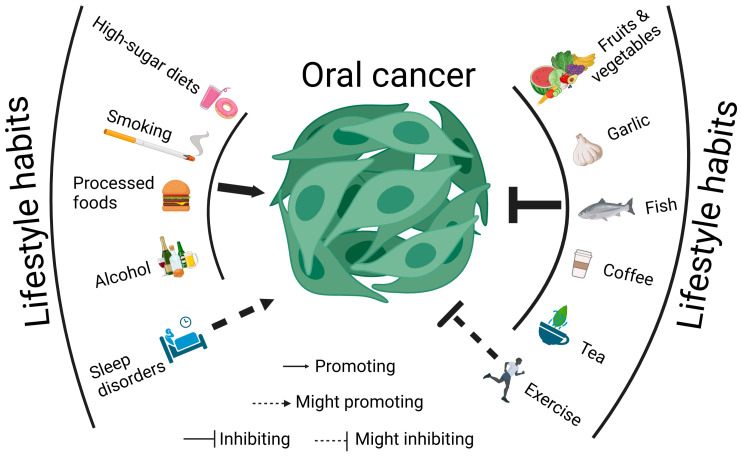
Oral cancer progression is affected by lifestyle habits.

The development and treatment of cancers, including oral cancer, can be affected by lifestyles, such as diet, smoking, exercise, and sleep. Healthy lifestyle habits can prevent oral cancer and benefit its treatment; in contrast, unhealthy habits may lead to the opposite effects (Figure 1 and Figure 2).

### 3.1. Diet

Diet, including food and drink, can impact the incidence, progression, and treatment of different types of cancers [59]. It significantly influences oral cancer, although the main etiologic factors of oral cancer are tobacco and alcohol [60]. About 10–15% of oral cancer cases are related to poor diet, which can increase inflammation [61,62]. Inflammation leads to oxidative stress; both can enhance each other [62]. Inflammation-induced reactive oxygen species (ROS) and reactive nitrogen species (RNS) trigger immune defense against injury and infection. However, excessive ROS and RNS can lead to chronic inflammation, which damages DNA and promotes mutations that lead to cancer development and benefit cancer progression [63]. An unbalanced diet challenges the immune system, stimulating oral cancer occurrence and progression and hindering its treatment. A meta-analysis study showed that pro-inflammatory diets with high dietary inflammatory index scores are associated with an increased risk of upper-aerodigestive tract cancers, including oral cancer [64].

#### 3.1.1. High-Sugar Diets Promote Oral Cancer Progression

Diets high in sugar stimulate chronic inflammation, contributing to cancer development [65]. Based on a large prospective study, sugary drink consumption was positively associated with the risk of developing overall cancer and breast cancer [66]. Sugar-free soft drinks are less cariogenic than sugar-containing ones [67]. Based on the analyses of three prospective United States (US) cohort studies, excessive sugar consumption can lead to the development of cancers, such as breast cancer, colorectal cancer, pancreatic cancer, and esophageal adenocarcinoma [68]. In a cohort study including 414 patients with HNSCC, high intakes of total carbohydrate and total sugar correlated with increased risks of all-cause and HNSCC-specific mortalities, and an increased risk of all-cause mortality was also associated with high intakes of glycemic load and simple carbohydrates [69].

#### 3.1.2. Natural Diets Could Combat Oral Cancer

Natural diets such as fruits, vegetables, beans, fish, nuts, and whole grains can combat cancer. The increased consumption of fruits and vegetables is inversely associated with OSCC frequency [70]. Diets lacking fruits and vegetables fail to provide essential micronutrients (e.g., iron and vitamin B12) and antioxidants, leaving cells vulnerable to oxidative damage and the development of oral cancer. Natural diet products, such as turmeric (curcumin), ginger, saffron, garlic, cinnamon, fenugreek, basil, Indian pennywort, tomato (lycopene), and green tea, which are used in daily life in South and South-East Asia, could help prevent oral cancer [71]. A meta-analysis reported that eating fish could reduce the risk of oral cancer in European populations [72]. The garlic extract component S-allylcysteine can inhibit tumor growth and epithelial–mesenchymal transition (a process facilitating cancer cell mobility, invasion, and metastasis) in a xenograft mouse model of oral cancer [73]. It also inhibits the proliferation of human oral cancer cells [74]. These observations suggest that garlic can combat oral cancer.

#### 3.1.3. Tea and Coffee Could Combat Oral Cancer

Tea and coffee can impact oral cancer progression. The increased consumption of tea, especially green tea, is inversely associated with OSCC frequency [19,70]. Coffee intake is also negatively associated with oral cancer risk [19,75,76]. A prospective US cohort study of 968,432 participants reported that drinking more than four cups of caffeinated coffee per day was associated with a 49% lower risk of oral/pharyngeal cancer death compared to no or occasional coffee intake; in contrast, no association of tea drinking with oral mortality was observed [77].

#### 3.1.4. Excessive Alcohol Consumption Promotes Oral Cancer Progression

Excessive alcohol consumption is a well-established major risk factor for oral cancer. Alcohol acts as a direct carcinogen and exacerbates the harmful effects of tobacco by increasing mucosal permeability to carcinogens. Many epidemiological studies have shown that alcohol consumption is strongly associated with an increased risk of oral and pharyngeal cancer [78]. However, alcohol consumption may have a beneficial effect on health under certain conditions. For example, low levels of alcohol consumption might protect against *Candida*-induced oral carcinogenesis and oral cancer progression [79].

#### 3.1.5. Processed Foods Could Promote Oral Cancer Progression

Processed foods promote systemic inflammation, creating an environment conducive to cancer development. Processed foods often contain carcinogenic compounds such as nitrosamines and acrylamides, which can damage cellular DNA [80]. The high consumption of red meat and thermally processed meat increases oral cancer risk [81]. The excessive consumption of salty meats, dairy, sausages, and fried and spicy foods is positively associated with OSCC frequency [70].

#### 3.1.6. Micronutrients Could Combat Oral Cancer

Micronutrients, particularly antioxidants such as vitamins A, C, and E, are critical in preventing DNA damage and neutralizing free radicals [82], protecting against oral cancer. Based on a study of 65 patients with oral cancer and 85 matched controls, low glutathione (GSH) levels and mild iron deficiency were associated with increased oxidative stress and oral cavity cancer risk [83], indicating that the naturally occurring antioxidant GSH and optimal levels of iron could combat oral cancer. Vitamin D deficiency is associated with increased oral cancer risk [84]. The active metabolite of vitamin D, calcitriol, can be used to prevent and treat several cancers, including OSCC [85]. Increased dietary fiber or vitamin C intake was associated with a decreased incidence of oral cancer [86]. Vitamin E, as an antioxidant, can prevent oral cancer at a very early stage, improve the effects of cancer chemotherapy, and reduce the side effects of chemotherapy and radiotherapy [87].

#### 3.1.7. Diet and TME

The TME is a complex local system surrounding a tumor, composed of cancer cells, the extracellular matrix, blood vessels, immune cells, fibroblasts, and other molecules, providing nutrients, oxygen, and a protective environment for cancer cells [88]. It plays a pivotal role in cancer progression. Diet significantly influences this microenvironment by modulating inflammation, immune response, and metabolic pathways. High-fat diet-induced obesity increased CD11b^+^Gr1^+^ myeloid-derived suppressor cells in the local immune microenvironment, promoting OSCC initiation and progression [89]. Fraga et al. reported that the immunomodulation of T helper cells by TME involved the vitamin D signaling pathway in OSCC [90]. In contrast, diets rich in omega-3 fatty acids, found in fatty fish and flaxseeds, can reduce inflammation and suppress tumor growth [91]. Conversely, diets high in saturated fats and refined carbohydrates create a pro-inflammatory microenvironment that accelerates tumor progression.

In India, traditional dietary practices, such as betel quid chewing, further modify the TME by introducing carcinogenic substances directly into the oral cavity [92,93,94]. In Canada and the US, high alcohol and processed food consumption similarly impact the TME, albeit through different mechanisms [80,94,95].

Therefore, dietary factors affect oral cancer progression differently. High-sugar diets, some processed foods, and excessive alcohol consumption promote oral cancer progression; in contrast, tea and coffee intake can suppress it (Figure 2).

### 3.2. Smoking

Smoking is a well-known major risk factor for OSCC. The use of tobacco can double the risk of developing head and neck cancer (HNC) [96]. Smoking and smokeless tobacco are associated with human buccal cell changes, such as micronuclei, bacterial adherence, genetic mutations, DNA polymorphisms, carcinogen–DNA adducts, and chromosomal abnormalities, which correlate with oral cancer occurrence [97]. Tobacco and alcohol can synergistically increase oral cancer risk [67]. Furthermore, smoking can hinder oral cancer treatment. It has detrimental effects on treatment efficacy, the acquisition of secondary malignancies, duration of survival, and quality of life in patients with cancer [98]. Continued smoking is associated with lower overall survival and locoregional control and a higher incidence of late toxicities in patients with HNC and undergoing radiotherapy [99]. In contrast, smoking cessation is associated with improved outcomes regarding overall survival, disease recurrence rates, and second primary tumors in patients with newly diagnosed HNC [100]. E-cigarettes (vapes or vaping) have become a rising trend in recent years. They contain carcinogenic components that induce DNA strand breaks and gene dysregulation; however, their role as an oral cancer risk factor remains unclear due to the lack of long-term, large-scale case–control studies [101].

### 3.3. Exercise

Physical activity has a strong association with reduced risks of breast, colon, endometrial, esophagus (adenocarcinoma), gastric, bladder, and renal cancers [102]; however, there is a lack of strong evidence to support its inverse association with the risk of HNC [102,103,104,105]. Patients with HNC who met the World Health Organization (WHO) physical activity guidelines reported less fatigue, better right hip flexor muscle strength, and better quality of life than those who did not have any physical activity [104], suggesting that physical activity can benefit these patients.

Physical activity interventions also benefit patients with HNC during and after treatment [106]; these patients could benefit more from combining aerobic and resistance training exercises than a single full-body exercise in counteracting physical decline and controlling anticancer therapy-associated symptoms [107].

### 3.4. Sleep

Sleep disorders, such as insomnia, parasomnia, and obstructive sleep apnea, are associated with diseases, including cancer. A study based on two million data points from the National Health Insurance System of Taiwan reported that patients with parasomnia had a significantly higher risk of developing oral cancer than those without parasomnia [108]. On the other hand, sleep disorders can be a consequence of cancer [109]. Sleep disorders are highly prevalent among patients with HNC before, during, and after treatment [110,111]. Insomnia and obstructive sleep apnea are associated with worse quality of life and oral mucositis in patients with HNC undergoing radiation therapy [111].

## 4. Oral Microbiome and Oral Cancer

The oral microbiome is composed of more than 700 bacterial species [112]. In recent years, many reviews have extensively summarized the relationship between oral microbiome and oral cancer [15,112,113,114,115,116,117,118]. The main finding is that oral microbiome dysbiosis (an imbalance in the composition of oral microorganisms) triggers chronic inflammation, which might contribute to oral cancer development. Oral microbiome dysbiosis can lead to the diagnosis of oral cancer and the development of new strategies for its treatment. The current understanding in this field mainly depends on DNA and RNA (e.g., 16S ribosomal RNA) sequencing, and most studies reported a correlation/association, but not a causal relationship, between oral microbiome and oral cancer development. It remains largely unknown which types of microbiome promote or suppress oral cancer development. Bacteria genera, such as *Fusobacterium* and *Treponema*, and the fungus *Candida albicans* [117,118], may be associated with OSCC occurrence. Human papillomavirus (HPV) can induce oropharyngeal squamous cell cancer (OPSCC) because HPV16 can integrate into the host genome and activate oncogenes [117,119,120]. In contrast, probiotic microbiomes can help combat oral cancer [121,122]. Future studies need to provide evidence to specify types of oral microbiome that benefit or suppress oral cancer progression using experimental models, such as genetically engineered mouse models [123]. Therefore, the oral microbiome could promote or suppress oral cancer progression, depending upon its composition (Figure 1).

## 5. Socioeconomic Factors and Oral Cancer

Socioeconomic status (SES) plays a significant role in the development and outcomes of oral cancer. Global trends in oral cancer illustrate the pervasive impact of SES, such as accessibility to healthcare, economic status, and education levels.

### 5.1. Inaccessibility to Healthcare Contributes to Delayed Diagnosis and Ineffective Treatment

Access to healthcare is compromised in populations with lower SES due to barriers such as lack of dental and health insurance, inability to make co-payments, inadequate transportation, and discrimination experience [124,125], leading to delayed diagnosis and suboptimal treatment outcomes of oral cancer. For example, Indigenous populations in Canada and rural communities in India highlight the urban–rural divide in healthcare delivery and cancer outcomes [126,127]. Rural populations often rely on under-resourced healthcare facilities in India, where oral cancers are diagnosed at advanced stages [128]. Similarly, in Canada, Indigenous populations and individuals in remote areas face significant challenges in accessing specialized care, resulting in poorer outcomes [129]. Moreover, systemic biases in healthcare delivery can result in inequitable treatment for marginalized groups [130].

### 5.2. Economic Status Affects Oral Cancer Diagnosis and Treatment

Low-income populations are disproportionately affected by poor treatment outcomes and increased risks of oral cancer [124,126]. They may lack the resources to afford timely interventions, follow-up care, and supportive therapies [131]. Further complicating this issue, most cases of oral cancer are asymptomatic until they reach advanced stages. Epidemiological studies have shown that regions with high poverty rates have correspondingly high oral cancer incidences. In Canada, while oral cancer rates are lower overall, the burden disproportionately affects First Nations Indigenous populations and those living in rural areas [132], whose incomes are relatively lower than non-Indigenous populations, according to Statista (www.statista.com (accessed on 7 March 2025)). Studies have reported significantly higher oral cancer mortality rates among Indigenous Canadians and later diagnosis compared to the national average, emphasizing the need for targeted interventions [132]. A significant increase in HNC has been observed in England, with cases rising from 10,735 in 2019 to over 11,000 in 2021. Deaths from these cancers also increased, reaching 3469 in 2020. The surge is largely attributed to factors such as smoking, alcohol consumption, and HPV infections. Notably, individuals residing in poorer areas are almost twice as likely to be diagnosed with HNC compared to those in wealthier regions [133].

### 5.3. Education Levels Affect Oral Cancer Diagnosis and Treatment

A recent study on public awareness and knowledge of oral cancer reported a significant lack of oral cancer awareness in 13 Middle Eastern and North African countries, especially in the population with lower levels of education and those who used tobacco [134], which might lead to delayed diagnosis and a widespread incidence of oral cancer. A lack of support systems due to conflicting family and work responsibilities and lower education levels can lead to poor adherence to treatment protocols and follow-up care [124,125]; this is reflected in data from the National Institute of Dental and Craniofacial Research, which show that the five-year survival rate of patients with oral cancer is lowest among American Indians/Alaska Natives and non-Hispanic Black populations across all stages, including localized lesions and metastases. A retrospective cohort study that analyzed 500 patients treated for oral cavity cancer between 2013 and 2019 revealed that patients with lower educational levels had a significantly higher risk of developing oral cavity cancer and exhibited a higher prevalence of risk factors such as smoking and alcohol consumption. The study also identified significant differences in overall survival rates based on educational attainment and sex, with lower survival observed among patients with less education [135].

### 5.4. Reflecting Global Inequalities

SES functions as a cause and a consequence of systemic injustice. Limiting access to healthcare, education, and resources perpetuates a cycle of vulnerability that drives up oral cancer risk. Therefore, low SES contributes to a high risk of oral cancer (Figure 1). Data from the WHO European Region indicate that Eastern European countries experience some of the highest prevalence rates of major oral diseases globally. The region reported that 50.1% of the adult population had a major oral disease in 2019, with caries of permanent teeth affecting 33.6% of the population. These high prevalence rates are often linked to socioeconomic factors, including limited access to dental care and lower health literacy [136]. A comprehensive review of European studies on socioeconomic inequalities in cancer incidence found that adults with low SES have an increased risk of HNC. The review highlighted that these disparities are more pronounced among men compared to women. The inequalities can be partially explained by lifestyle-related factors, notably smoking, which is more prevalent in lower socioeconomic groups [137]. The impact of SES on survival rates underscores the importance of recognizing the intersectionality of risk factors when analyzing survival outcomes [124,125]. A nationwide study in Germany investigating socioeconomic inequalities in cancer mortality indicated substantial disparities, with lower SES associated with higher cancer mortality rates. These findings underscore the need for targeted cancer prevention and control strategies in socioeconomically disadvantaged regions [138]. Addressing SES disparities requires a multifaceted approach, including public health campaigns, equitable healthcare policies, and community-based interventions. These findings highlight the importance of considering socioeconomic factors in the etiology of oral cancer [93]. Public health measures, including early diagnosis and prevention, should target individuals with lower SES, as they face higher risk. The WHO Commission on Social Determinants of Health should address some of these barriers to healthcare access, particularly for populations with low SES [93].

## 6. Oral Cancer Is Affected by Crosstalk Between Different Factors

**Figure 3:**
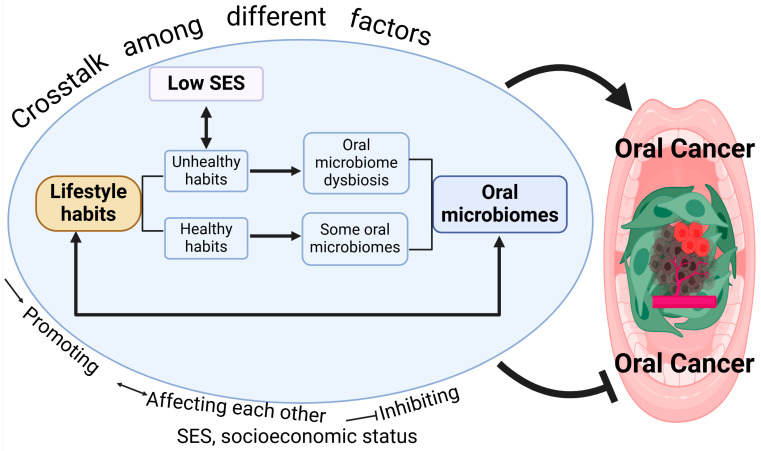
Oral cancer is affected by crosstalk among different factors.

Oral cancer arises from a combination of genetic, environmental, and lifestyle factors. Multiple factors can affect its progression individually (Figure 1) and via crosstalk between each other (Figure 3).

### 6.1. SES and Lifestyle Habits

Low SES directly correlates with increased exposure to oral cancer risk factors, such as smoking, excessive alcohol consumption, and poor oral hygiene practices [139]. Oral cancer progression is affected by the associations among different factors. Oral cancer outcomes differ starkly between developed countries, such as Canada and the US, and developing countries, such as India, which have differences in SES. In India, oral cancer constitutes one-third of the global burden and accounts for 30% of all cancers, predominantly affecting individuals from lower socioeconomic backgrounds who engage in risky behaviors, such as tobacco and betel quid use, with approximately five deaths occurring every hour [127,128,140,141]. HPV is responsible for up to 72% of HNSCC in developed nations [4]. Canada reports low overall rates but faces increasing HPV-related oropharyngeal cancers [142]. Black Americans with oral cancer have a five-year survival rate of just 50%, largely due to late-stage diagnoses and a history of tobacco and alcohol use [124,125].

Tobacco is a leading cause of oral cancer, particularly in regions such as India [140]. In developing regions, betel quid chewing is an endemic habit dramatically elevating oral cancer incidence [127]. Excessive alcohol consumption further compounds the risk, as alcohol acts as a solvent for carcinogens and promotes mucosal irritation. In Canada and the US, while smoking rates have declined due to stringent public health policies, alcohol use remains a prevalent risk factor, especially in Indigenous and rural populations [94]. In the US, racial disparities compound SES inequalities. Non-Hispanic Black males, historically at high risk for oral cancer, have shown declining rates, but low-income populations continue to face limited access to early diagnosis and treatment [143]. Poor oral hygiene is another critical factor tied to SES. Limited access to basic dental care, combined with a lack of education on oral health maintenance, exacerbates the risk of oral cancer. Chronic inflammation caused by poor oral hygiene creates a conducive environment for carcinogenesis [144].

Dietary patterns reflect socioeconomic and cultural influences. In India, traditional diets are often rich in spices and low in fruits and vegetables, particularly in lower-income households [145]. This nutritional imbalance contributes to increased susceptibility to oral cancer. In Canada, while the general population has better access to diverse foods, marginalized groups, including Indigenous peoples, often consume diets high in processed and sugary foods due to economic constraints and food insecurity [146], contributing to a higher incidence of oral cancer.

Socioeconomic factors shape the environment in which lifestyle habits develop. Due to limited access to healthcare, nutrition, and educational resources, individuals with very low incomes are more likely to engage in high-risk behaviors such as smoking, alcohol consumption, and poor dietary choices. Conversely, unhealthy lifestyle habits may negatively affect SES, creating a vicious cycle for the management and development of oral cancer. The convergence of these factors amplifies oral cancer risk, illustrating the need for interventions that address both socioeconomic conditions and behavioral modifications.

### 6.2. Lifestyle Habits and Oral Microbiome

Changes in the oral microbiome due to poor hygiene or dietary deficiencies can disrupt cellular pathways, facilitating tumor initiation and progression [147]. Westernized diets containing farmed animal meats, high-sugar dairy products, refined vegetable oils, and processed grains are associated with increased acid-producing and acid-tolerant organisms in oral microbiota [148]. Acid-producing and acid-tolerant bacteria could promote or disadvantage oral cancer development and treatment, depending on the bacterial types [149].

Smoking can lead to oral microbiome dysbiosis [150]. Alcohol consumption alters oral microbial diversity and composition [151]. Future studies are needed to clarify the impact of smoking- and alcohol-induced oral microbiome dysbiosis on oral cancer.

While lifestyle factors such as poor diet, smoking, and alcohol consumption are recognized as significant risk factors for oral cancer, their influences go beyond behavior—they actively shape the microenvironment of the oral cavity, which may change the composition of the oral microbiome. Green tea could play a chemopreventative role against oral cancer [152]. Its liquid consumption can alter the human oral microbiome and reduce the abundance of functional pathways relevant to carcinogenesis [153].

A recent study showed that intensive physical activity did not change the diversity of oral microbiota but reduced the number of bacterial species [154]. Furthermore, intensive physical activity increases the abundance of butyrate and succinate-producing bacteria, which could benefit the host immune system’s microbiome homeostasis [154]. Oral bacterial counts markedly increase during sleep; sleep affects the microbiome in different locations in the oral cavity, and changes in the microbiome composition depend on the surface characteristics of oral biofilms [155]. Obstructive sleep apnea and related conditions might be associated with different oral microbial compositions [156]. Oral microbiome diversity can affect the association between sleep duration and depression [157]. The impact of the oral microbiome on other lifestyle habits needs to be studied.

## 7. Perspectives: Strategies for Oral Cancer Management and Treatment

While existing literature reviews have explored the impacts of individual factors on oral cancer, integrating multiple factors remains scarce. This review synthesizes these diverse dimensions to present a comprehensive framework for understanding and addressing oral cancer globally. By bridging fragmented knowledge, it advocates for innovative, unified solutions to combat this multifaceted disease.

Oral cancer is affected by multiple factors and their associations. Addressing it necessitates a holistic, multidisciplinary approach. Combining prevention strategies, such as public education, tobacco cessation, and dietary interventions, with early detection and advanced treatment modalities is imperative. A systems-level integration of healthcare, social support, and policymaking is essential to reduce SES disparities and improve outcomes [140].

Lifestyles, such as diet, drinking, smoking, exercise, and sleep, affect oral cancer in different ways. Some diets are controllable, offering significant potential for preventing and intervening in oral cancer; others might be difficult to control because they are often dependent on social determinants of health. Dietary habits play dual roles in the development and progression of oral cancer [147]. Poor diets, characterized by high sugar intake and low fruit and vegetable consumption, contribute to inflammation and oxidative stress, benefiting oral cancer development but not its treatment [158]; in contrast, protective dietary elements, such as antioxidants, can mitigate DNA damage and tumorigenesis [159]. Public health campaigns should emphasize the importance of balanced diets rich in fruits, vegetables, and whole grains, while reducing sugar and alcohol consumption. Low sugar, high amounts of fruits, vegetables, green tea, and antioxidant micronutrients, increased vitamin D levels, less alcohol and processed foods, no smoking, physical exercise, and good sleep could help prevent and fight oral cancer. Approaches targeting the TME can improve cancer therapy. For example, natural products, such as curcumin and resveratrol, can combat oral cancer by targeting TME and inducing cancer cell death [160].

## 8. Conclusions

Oral cancer is a multifactorial disease influenced by a combination of environmental, behavioral, genetic, and biological factors. Globally, well-established risk factors for oral cancer include tobacco use (both smoking and smokeless forms), excessive alcohol consumption, infection with high-risk HPV subtypes, chewing betel quid and areca nut, and poor oral hygiene. Dietary deficiencies, particularly the low intake of fruits, vegetables, and micronutrients (e.g., vitamins A, C, E), contribute to oxidative stress and mucosal vulnerability, enhancing cancer risk. Additionally, genetic predispositions—including mutations in tumor suppressor genes and oncogenes—and family history of cancer may influence susceptibility. Emerging evidence also implicates the dysbiosis of the oral microbiome, chronic inflammation, and immune evasion mechanisms, such as the overexpression of immune checkpoint proteins (e.g., PD-L1), in oral carcinogenesis. SES plays a pivotal role in shaping exposure to these risk factors, influencing health behaviors, access to care, and early diagnosis. Other contributing elements include occupational exposures (e.g., to wood dust and certain chemicals), radiation exposure, and systemic conditions such as immunosuppression.

While many factors contribute to the development of oral cancer, this review focuses on three key extracellular dimensions that reflect both established and emerging areas of research. It integrates multiple dimensions of oral cancer, linking socioeconomic factors, lifestyles, and the oral microbiome into a cohesive framework. These areas are examined, emphasizing their interplay and implications for public health, prevention, and therapeutic strategies. The review summarizes the impacts of individual factors and their interplay in oral cancer progression. Oral cancer is differentially affected by lifestyle habits, including diet, smoking, exercise, and sleep. Diets high in sugar, some processed foods, excessive alcohol consumption, and smoking promote oral cancer progression, whereas natural diets rich in fruits, vegetables, fish, and garlic, along with tea and coffee consumption, may have protective effects. Physical exercise may support cancer prevention, while sleep disorders could contribute to its progression; however, further studies are needed to confirm these associations. The oral microbiome plays a dual role, either promoting or suppressing oral cancer, depending on its composition and context. Despite increasing research, the specific microbiome profiles that contribute to or inhibit oral cancer remain unclear. Low SES is associated with increased oral cancer risk but does not aid in treatment outcomes. Furthermore, the interplay among SES, lifestyle habits, and microbiome forms a complex cycle—low SES contributes to unhealthy behaviors, and these behaviors can, in turn, perpetuate socioeconomic disadvantages. Additionally, lifestyle factors and oral microbiome composition can influence each other, with potential implications for sleep quality and cancer risk. This review has certain limitations. Due to the extensive body of literature on oral cancer, it was not possible to comprehensively cover all influencing factors. Additionally, we have focused on the interactions among different external contributors to oral cancer; some significant individual studies may not have been included.

Based on these findings, public health strategies should incorporate dietary modifications, smoking cessation programs, and targeted early screening efforts to reduce disparities in oral cancer outcomes. Future research should emphasize the intricate relationships between oral microbiome, lifestyle habits, SES, and genetic alterations to develop personalized medicine interventions. Emerging technologies, including artificial intelligence-driven diagnostic tools, precise medicine approaches, and microbiome-based therapies, hold promise for improving early detection and patient outcomes. Integrating digital health solutions, such as wearable biosensors for early cancer detection and telemedicine for equitable access to specialized care, may further enhance the quality of life and survival rates in patients with oral cancer. Bridging the gap between fundamental research and clinical practice through interdisciplinary collaboration will be crucial in advancing oral cancer prevention, treatment, and patient well-being.

## Data Availability

This is a review article, and the study did not generate any new data.

## Abbreviations

The following abbreviations are used in this manuscript:

*APC*	adenomatous polyposis coli
ATP	adenosine triphosphate
BCL-2	B-cell lymphoma 2
bFGF	basic fibroblast growth factor
*BNIP3*	BCL-2/adenovirus E1B 19-kDa-interacting protein 3
*BRAF*	V-Raf murine sarcoma viral oncogene homolog B
*CDKN2A*	cyclin-dependent kinase inhibitor 1A
*CTNNB1*	catenin beta 1
DNA	deoxyribonucleic acid
EGFR	epidermal growth factor receptor
GLUT1	glucose transporter type 1
GSH	glutathione
HER2	human epidermal growth factor receptor 2
HIF-1α	hypoxia-inducible factor-1α
HIF-2α	hypoxia-inducible factor-2α
HK2	hexokinase 2
HNC	head and neck cancer
HNSCC	head and neck squamous cell carcinoma
HPV	human oral papillomavirus
IL-10	interleukin-10
IL-8	interleukin-8
LDH-A	lactate dehydrogenase-A
M2-TAMs	M2-like tumor-associated macrophages
MDSC	myeloid-derived suppressor cell
MHC	major histocompatibility complex
MMP	matrix metalloproteinase
*MYB-NFIB*	myeloblastosis viral oncogene homolog–nuclear factor I/B
*NRAS*	neuroblastoma RAS viral oncogene homolog
OC	oral cancer
OPSCC	oropharyngeal squamous cell cancer
OSCC	squamous cell carcinoma
PD-1	programmed cell death protein 1
PDGF	platelet-derived growth factor
PD-L1	programmed cell death ligand 1
PKM2	pyruvate kinase M2
RAS	rat sarcoma virus
rDNA	ribosomal DNA
RNA	ribonucleic acid
RNS	reactive nitrogen species
ROS	reactive oxygen species
rRNA	ribosomal RNA
SES	socioeconomic status
TAMs	tumor-associated macrophages
TGF-β	transforming growth factor-beta
TILs	tumor-infiltrating lymphocytes
TME	tumor microenvironment
*TP53*	tumor protein p53
US	United States
VEGF	vascular endothelial growth factor
WHO	World Health Organization
